# Primary Stability of Dental Implants in Low-Density (10 and 20 pcf) Polyurethane Foam Blocks: Conical vs Cylindrical Implants

**DOI:** 10.3390/ijerph17082617

**Published:** 2020-04-11

**Authors:** Luca Comuzzi, Margherita Tumedei, Ana Emilia Pontes, Adriano Piattelli, Giovanna Iezzi

**Affiliations:** 1 Private Practice, San Vendemiano-Conegliano, 31020 Treviso, Italy; luca.comuzzi@gmail.com; 2 Department of Medical, Oral and Biotechnological Sciences, University “G. D’Annunzio” of Chieti-Pescara, 66100 Chieti, Italy; apiattelli@unich.it (A.P.); gio.iezzi@unich.it (G.I.); 3 Department of Dentistry, Federal University of Juiz de Fora-Campus Governador Valadares, São Paulo 01000, Brazil; anaemiliapontes@yahoo.com.br; 4 Biomaterials Engineering, Catholic University of San Antonio de Murcia (UCAM), 30001 Murcia, Spain; 5 Fondazione Villaserena per la Ricerca, Città Sant’Angelo, 65121 Pescara, Italy

**Keywords:** bone density, cylindrical implants, conical implants, implant stability quotient, insertion and pull-out torque, polyurethane foam blocks, primary stability

## Abstract

Background: The aim of the present study was to compare, in low-density polyurethane blocks, the primary implant stability values (micromobility) and removal torque values of three different implant geometries in two different bone densities representing the structure of the human posterior jaws. Methods: A total of 60 implants were used in the present investigation: twenty implants for each of three groups (group A, group B, and group C), in both polyurethane 10 pcf and 20 pcf densities. The insertion torque, pull-out torque, and implant stability quotient (ISQ) values were obtained. Results: No differences were found in the values of Group A and Group B implants. In both these groups, the insertion torques were quite low in the 10 pcf blocks. Better results were found in the 20 pcf blocks, which showed very good stability of the implants. The pull-out values were slightly lower than the insertion torque values. High ISQ values were found in Group A and B implants. Lower values were present in Group C implants. Conclusions: The present investigation evaluated implants with different geometries that are available on the market, and not experimental implants specifically created for the study. The authors aimed to simulate real clinical conditions (poor-density bone or immediate post-extraction implants) in which knowledge of dental implant features, which may be useful in increasing the primary stability, may help the oral surgeon during the surgery planning.

## 1. Introduction

Oral rehabilitation with dental implants represents a highly predictable procedure for partial and full edentulism, characterized by a success rate of over 90% [1]. The osseointegration of dental implants is determined by two different processes: the primary stability obtained with the mechanical engagement of the screw with the bone wall of the preparation site, and the secondary stability, due to new bone formation during the healing period [2,3].

The relationship between these two conditions is influenced by the absence of micromovements of the implant after its placement into the preparation site [3].

Implant stability is influenced by several factors: bone density [4], implant macro- and micro-geometry [5,6], and surgical technique [7,8,9]. In the case of poor bone quality, or immediate post-extraction implants, the primary stability could be improved by the selection of a specific implant geometry and thread design.

For this reason, it is important to compare the primary stability of different implant geometries in order to establish which implant shape or geometry could be useful for obtaining high primary fixation in each clinical condition.

Several factors could condition local bone density: sex, age, medical treatments, systemic diseases, but, generally, the posterior regions of the maxilla are clinically characterized by a lower density of bone tissue [10,11,12].

Many different non-invasive tests have been proposed to evaluate, clinically and in laboratory conditions, the primary stability of dental implants.

The resonance frequency analysis (RFA) is a repeatable technique for implant stability evaluation that provides a measurement of the micromovements of the implant positioned in the bone site [13,14,15,16].

On the contrary, the insertion and removal torque evaluation is a non-repeatable measurement of the mechanical friction between an implant and bone walls during implant insertion and unscrewing [16,17].

With the use of polyurethane block sheets, it is possible to standardize the mechanical response to the forces generated during implant positioning [18].

In fact, the in vitro simulation on polyurethane is able to overcome the anatomical limits of implant osteotomies performed on ex-vivo samples, such as human cadaveric bone [19], bovine and pig ribs [19,20,21], and rabbit tibiae [22].

The human cadaver bone or fresh animal bone shows quality modifications over time, and selecting various bone blocks of the same quality represents an operation with high variability. To overcome the possible biases due to differences in bone quality, it is possible to use synthetic bony blocks made of rigid polyurethane [23,24,25]. The uniformity and consistent properties of rigid polyurethane foam make it an ideal material for comparative mechanical tests of different implant designs.

Moreover, as reported in the literature, polyurethane blocks are commercially available in different densities and thicknesses, and as such, are able to simulate the different consistencies of bone tissues present in the different regions of the upper and lower jaws [13,14,26].

The aim of the present study was to compare, in low-density polyurethane blocks, the primary implant stability values (micromobility) and removal torque values of three different implant geometries in two different bone densities representing the structure of the human posterior jaws.

## 2. Materials and Methods

### 2.1. Implants

**Figure 1:**
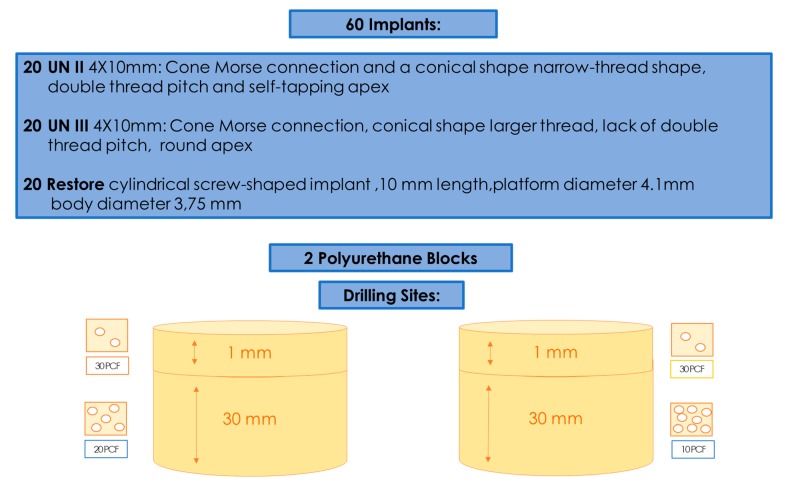
Summary of the study design of the present investigation.

**Figure 2:**
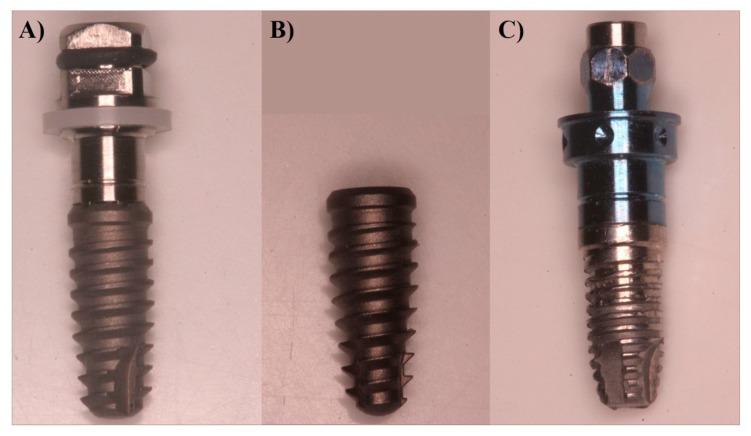
Up close photographs showing the details of the implants investigated. (**A**) UNII. (**B**) UNIII. (**C**) RBM Restore.

In total, 60 implants were used, divided into three groups: Group A had 20 implants (4 mm diameter and 10 mm length) with a Cone Morse connection and a conical shape (UN II, Implacil De Bortoli, Sao Paulo, Brasil); Group B also had 20 implants (4 mm diameter and 10 mm length) with a Cone Morse connection and a conical shape (UN III, Implacil De Bortoli, Sao Paulo, Brasil) (Figure 1 and Figure 2A,B); and Group C had 20 (4.1 mm × 10 mm length) cylindrical screw-shaped implants (RBM Restore, Keystone Dental, Burlington, MA, USA) (Figure 2C). The UN III macro design differed from the UN II implants regarding its larger thread, the lack of double thread pitch, a round, not self-tapping, apex, and the presence of healing chambers between the cutting surface of the threads. The UN II presented a narrow-thread shape, double thread pitch, and self-tapping apex. The surface treatment used was performed by blasting with microparticles (~100 μm) of titanium dioxide and followed by double etching with maleic acid. The surfaces of the Group C implants were sandblasted with 150 microns hydroxyapatite particles (Figure 1).

### 2.2. Polyurethane Blocks

The American Society for Testing and Materials (ASTM F-1839-08) has approved the use of polyurethane and has recognized it as a standard for testing instruments and oral implants for comparative testing of bone screws (“Standard Specification for Rigid Polyurethane Foam for Use as a Standard Material for Test Orthopedic Devices for Instruments”). Different types of solid rigid polyurethane blocks, which were 120 mm × 170 mm × 31 mm foam blocks (SawBones H, Pacific Research Laboratories Inc., Vashon, WA, USA) with homogeneous densities, were selected for the present investigation. The densities of polyurethane foam were 10 pounds per cubic foot (pcf) and 20 pcf. A 1-mm thin layer of 30 pcf was added to all of these blocks in order to simulate real clinical conditions.

### 2.3. Implant Insertion

**Figure 3:**
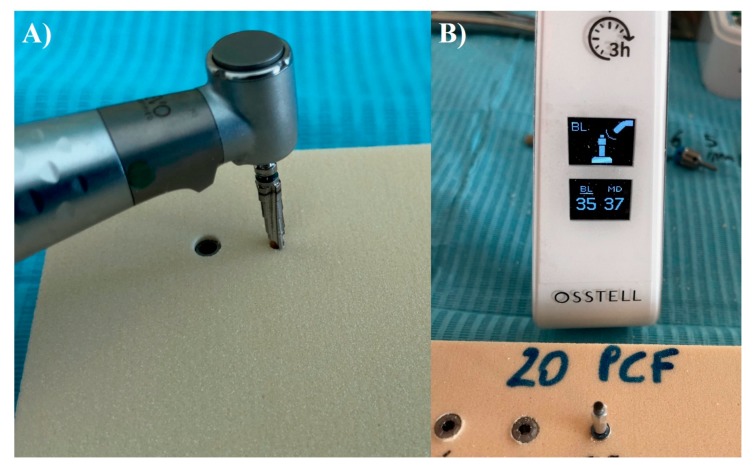
**(A**) Preparation of the implant site into the 10 pcf polyurethane block. (**B**) Resonance frequency analysis (RFA) micromovement measurement of the implant positioned into the block.

Group A and B implants were inserted using a lance drill, then a 2-mm bur at 1200 Rpm, and subsequently a conical 3.5-mm bur at 800 rpm with the implant insertion at 20 rpm. Group C implants were inserted following the protocol of the manufacturer by using an implant lance drill, a 2-mm drill (1600 rpm), and a 3-mm final drill (800 rpm). The handpiece was calibrated at a speed of 70 rpm and a torque of 30 Ncm. (Figure 3A,B). Torque values were taken with software (ImpDat Plus, East Lansing, MI, USA) installed on a digital card.

### 2.4. Insertion Torque and Removal Torque

The insertion torque (IT, Ncm) values indicated the force of the maximum clockwise movement that stripped bone. The investigation was conducted by a single operator (LC) who compared the torque insertion and the removal strength values of the Group A, B, and C implants. The study was conducted comparing the insertion torque and the removal strength values with a calibrated torque meter with a torque range of 5–80 N/cm. The final 1 mm insertion torque of the implants into the bone sheets was recorded. In the present study, mechanical torque gauges (Implacil De Bortoli, Sao Paulo, Brasil) were used to assess the insertion torque and the removal strength values.

### 2.5. Resonance Frequency Analysis

After implant insertion, primary stability was measured using resonance frequency analysis (RFA) values expressed in ISQ with hand-screwed Smart-Pegs (number 7 for group A and B implants and number 1 for the group C implants) (Osstell Mentor Device, Integration Diagnostic AB, Savadelen, Swden) (Figure 3B). The implant stability quotient (ISQ) ranged from 0 to 100 (measured between 3500 and 85,000 Hz) and was divided into low (<60 ISQ), medium (60–70 ISQ), and high stability (>70 ISQ). For each specimen, the RFA measurement was repeated two times. Measurements were performed in two orientations separated by a 90-degree angle, and the average ISQ values were calculated.

### 2.6. Statistical Evaluation

The normal distribution of the data was evaluated by the Shapiro–Wilks test and the differences between the study groups regarding insertion torque, removal, and RFA groups were analyzed by one-way analysis of variance (ANOVA) followed by Tukey post-hoc test. A *p*-value < 0.05 was considered statistically significant. Data treatment and statistical analysis were performed by Excel origin (Microsoft Company, Redmond, WA, USA) and StatPlus 6 software (AnalystSoft, Walnut, CA, USA). The accuracy of RFA assessment was evaluated by Bland–Altman and linear regression model to determine the agreement between the ISQ measurements.

## 3. Results

**Figure 4:**
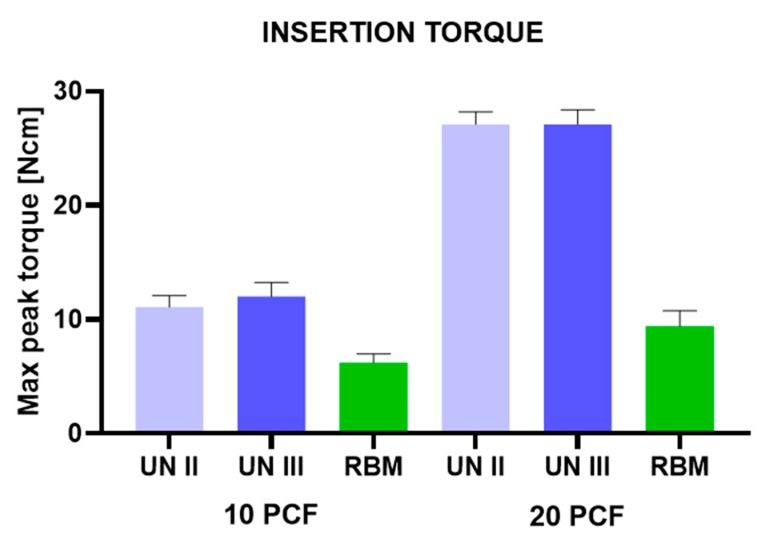
Insertion torque of UN II, UN III, and RBM implants in polyurethane foam blocks (10 pcf; 20 pcf).

No differences were found in the values of the Group A and Group B implants. Regarding the implants for both of these groups, the insertion torque values were quite low in the 10 pcf blocks (16–28 Ncm) (Figure 4, Table 1).

**Figure 5:**
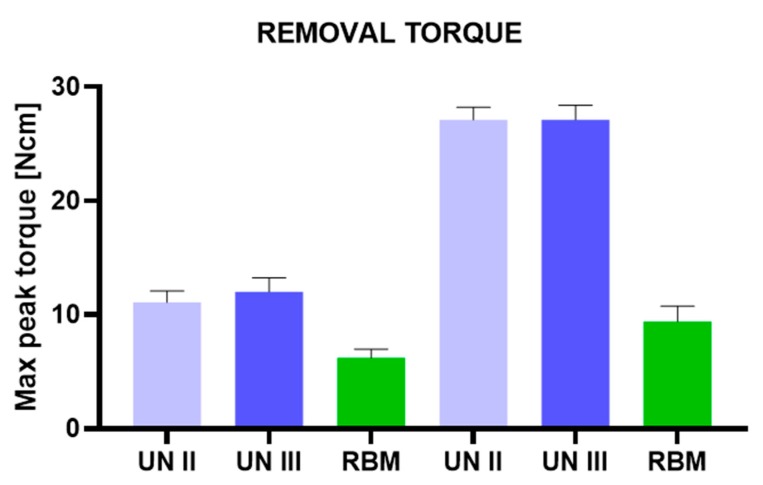
Removal torque of UN II, UN III, and RBM implants in polyurethane foam blocks (10 pcf; 20 pcf).

**Figure 6:**
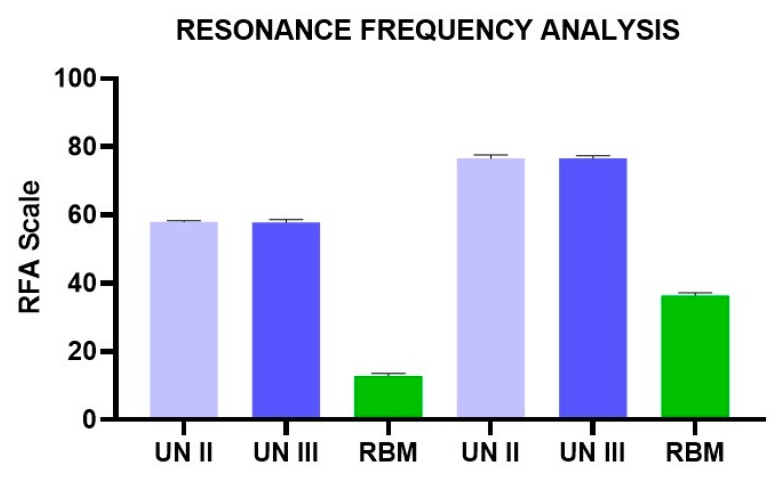
RFA of UN II, UN III, and RBM implants in polyurethane foam blocks (10 pcf; 20pcf).

Better results were found in the 20 pcf blocks, which showed very good stability of the implants. The removal values for both the Group A and B implants were slightly lower than the insertion torque values. High ISQ values were found in both the Group A and B implants (57–80). The Group C implants, on the other hand, presented very low insertion torque values (6–12 Ncm) in both polyurethane densities (10 and 20 pcf) (Figure 4; Table 1 and Table 2). Also, the removal values were very low (5–10 Ncm) (Figure 5; Table 3 and Table 4). Furthermore, the ISQ values were also in the very low range (10–37) (Figure 6; Table 5 and Table 6).

## 4. Discussion

Primary stability is defined as the mechanical engagement of the implant during positioning and represents an essential requirement for osseointegration [27].

The present investigation evaluated implants with different geometries that are present on the market and not experimental implants specifically created for the study. The authors aimed to simulate real clinical conditions (poor-density bone or immediate post-extraction implants) in which the knowledge of dental implant features, which may be useful in increasing the primary stability, could help the oral surgeon to plan the surgery.

Regarding the macro-design, a cylindrical shape could lead to a higher bone-to-implant contact percentage, if compared with a conical geometry, providing that local factors such as depth of positioning, bone density, and surgical technique are equal [16,18,28]. Micro-geometry, threads pitch, and surface roughness could also significantly influence the primary stability [5,29,30].

The use of a polyurethane model represents a cost-effective technique for evaluating the mechanical properties of dental implant protocols. This material could be tested in several different forms, such as solid foams [13,31], cellular rigid blocks [32], and blocks with composite densities [31,33,34], thus allowing researchers to analyze experimental measurements recorded on a structurally homogeneous substrate while overcoming the anatomical and ethical limits of ex vivo investigations.

Moreover, the present investigation tested a polyurethane density of 10 pcf and 20 pcf, equivalent, respectively, to 0.16 g/cm^3^ and 0.32 g/cm^3^, covered by a layer of cortical thickness to simulate the situation in the posterior region of the maxilla.

In the literature, only a few studies have evaluated dental implant primary stability on low-density double-layered polyurethane, which demonstrates the novelty of the present investigation; the authors have reason to believe that low-density double-layered polyurethane provides a simulation closer to clinical conditions encountered for surgeries on humans [35,36].

In fact, Devlin et al. reported in humans a mean bone mineral density of the posterior maxilla of 0.31 g/cm^3^ compared to the anterior maxilla, which showed a mean density of 0.55 g/cm^3^.

In the present study, the Group A and B implants showed high stability in low-density polyurethane blocks, with no statistical differences between the two groups, notwithstanding the diversities of the apex and thread profiles. Group C implants showed lower values.

Within the limitations of the present in vitro model, the investigation outcomes could be interpreted as a dominant effect of the implant macro-design (such as tapered and straight, conical and cylindrical geometry) rather than the effects that cutting chamber and apex profile played on IT and stability as a result of the screw positioning.

Markovic et al. reported in vivo an increased implant stability and RFA values in favor of self-tapping implants than non-self-tapping and round apex implants [37].

The treatment of low-density bone represents a procedure that requires high clinical sensibility and, possibly, as reported in the literature, also implant site under-preparation and condensation of the peripheral bone walls [7,38,39].

In the present study, the Group A and B implants showed a high level of IT and RFA on polyurethane blocks, including for 10 pcf blocks [40,41].

The use of conical shaped implants could represent an advantage in cases of reduced diameter alveolar ridges [42,43,44,45].

## 5. Conclusions

The present investigation evaluated implants with different geometries that are present on the market, and not experimental implants specifically created for the study. The authors aimed to simulate real clinical conditions (poor-density bone or immediate post-extraction implants) in which the knowledge of dental implant features, which may be useful for increasing the primary stability, may help the oral surgeon during the surgery planning.

## Figures and Tables

**Table 1:** Summary of the insertion torque values of the different experimental Groups.

Insertion Torque	10 PCF			20 PCF		
	UN II (A)	UN III (B)	RBM (C)	UN II (D)	UN III (E)	RBM (F)
Mean	17.00	16.80	7.00	29.10	31.10	13.30
Std. Deviation	0.94	1.14	1.05	0.99	0.99	1.16

**Table 2:** Insertion torque ANOVA Bonferroni post hoc groups comparison.

Multiple Comparison Insertion Torque	Mean Diff	95.00% CI of Diff	Adjusted *p* Value
**A-B**	0.20	−1.156 to 1.556	>0.9999
B-C	9.80	8.444 to 11.16	<0.0001
A-C	10.00	8.644 to 11.36	<0.0001
D-E	−2.00	−3.356 to −0.6439	0.0007
E-F	17.80	16.44 to 19.16	<0.0001
D-F	15.80	14.44 to 17.16	<0.0001
A-D	−12.10	−13.46 to −10.74	<0.0001
B-E	−14.30	−15.66 to −12.94	<0.0001
C-F	−6.30	−7.656 to −4.944	<0.0001

**Table 3:** Summary of the removal torque values of the different experimental groups.

Removal Torque	10 PCF			20 PCF		
	UN II (A)	UN III (B)	RBM (C)	UN II (D)	UN III (E)	RBM (F)
**Mean**	11.10	12.00	6.20	27.10	27.10	9.40
**Std. Deviation**	0.99	1.25	0.79	1.10	1.29	1.35

**Table 4:** Removal ANOVA Bonferroni post hoc groups comparison.

Multiple Comparison Removal	Mean Diff	95.00% CI of Diff	Adjusted *p* Value
A-B	−0.90	−2.378 to 0.5782	0.7585
B-C	5.80	4.322 to 7.278	<0.0001
A-C	4.90	3.422 to 6.378	<0.0001
D-E	0.00	−1.478 to 1.478	>0.9999
E-F	17.70	16.22 to 19.18	<0.0001
D-F	17.70	16.22 to 19.18	<0.0001
A-D	−16.00	−17.48 to −14.52	<0.0001
B-E	−15.10	−16.58 to −13.62	<0.0001
C-F	−3.20	−4.678 to −1.722	<0.0001

**Table 5:** Summary of the RFA values of the different experimental groups.

RFA	10 PCF			20 PCF		
	UN II (A)	UN III (B)	RBM (C)	UN II (D)	UN III (E)	RBM (F)
Mean	58.05	57.90	12.85	76.65	76.60	36.40
Std. Deviation	0.28	0.81	0.78	1.00	0.77	0.77

**Table 6:** RFA ANOVA Bonferroni post hoc groups comparison.

Multiple Comparison RFA	Mean Diff	95.00% CI of Diff	Adjusted *p* Value
A-B	−2.25	0.15	>0.9999
B-C	20.80	45.05	<0.0001
A-C	0.15	45.20	<0.0001
D-E	18.70	0.05	>0.9999
E-F	20.95	40.20	<0.0001
D-F	18.55	40.25	<0.0001
A-D	−1.55	−18.60	<0.0001
B-E	3.35	−18.70	<0.0001
C-F	0.05	−23.55	<0.0001
